# Gut Inflammation in Association With Pathogenesis of Parkinson’s Disease

**DOI:** 10.3389/fnmol.2019.00218

**Published:** 2019-09-13

**Authors:** Qian-Qian Chen, Caroline Haikal, Wen Li, Jia-Yi Li

**Affiliations:** ^1^Institute of Neuroscience, College of Life and Health Sciences, Northeastern University, Shenyang, China; ^2^Neural Plasticity and Repair Unit, Wallenberg Neuroscience Center, Department of Experimental Medical Science, Lund University, Lund, Sweden; ^3^Institute of Health Sciences, China Medical University, Shenyang, China

**Keywords:** gut inflammation, Parkinson’s disease, α-synuclein, oxidative stress, cyclooxygenase-2, glutamate excitotoxicity, T-cell

## Abstract

Parkinson’s disease (PD) is a neurodegenerative disease that is generally thought to be caused by multiple factors, including environmental and genetic factors. Emerging evidence suggests that intestinal disturbances, such as constipation, are common non-motor symptoms of PD. Gut inflammation may be closely associated with pathogenesis in PD. This review aims to discuss the cross-talk between gut inflammation and PD pathology initiation and progression. Firstly, we will highlight the studies demonstrating how gut inflammation is related to PD. Secondly, we will analyze how gut inflammation spreads from the gastro-intestine to the brain. Here, we will mainly discuss the neural pathway of pathologic α-syn and the systemic inflammatory routes. Thereafter, we will address how alterations in the brain subsequently lead to dopaminergic neuron degeneration, in which oxidative stress, glutamate excitotoxicity, T cell driven inflammation and cyclooxygenase-2 (COX-2) are involved. We conclude a model of PD triggered by gut inflammation, which provides a new angle to understand the mechanisms of the disease.

## Introduction

Parkinson’s disease (PD) is a neurodegenerative disease that is characterized by the degeneration of dopaminergic neurons in the substantia nigra pars compacta (SNpc) (Hostiuc et al., 2016) and the presence of Lewy bodies (LBs) (Engelhardt, 2017), in which the primary protein component is misfolded and aggregated α-synuclein (α-syn) (Spillantini et al., 1998). PD patients typically exhibit motor symptoms, such as tremor, stiffness, unstable posture, and slowness of movement (Jankovic, 2008), which are accompanied, and often preceded by a series of non-motor symptoms, such as intestinal dysfunction (Mukherjee et al., 2016), sleep disorders (Partinen, 1997; Ciric et al., 2018), depression (Taylor et al., 1986) and cognitive impairment (Starkstein et al., 1989). Among these non-motor symptoms, intestinal dysfunction has been paid special attention to, not only because it often appears prior to the motor symptoms (Suzuki et al., 2019), but also because α-syn aggregates have been detected in the gastrointestinal tract years before the motor-symptom onset (Cersosimo, 2015; Stokholm et al., 2016; Kim et al., 2017; Lu et al., 2017). LBs present in the gastrointestinal tract were first described in 1986 by Qualman et al. (1984) in which LBs were detected in the esophagus and colon in 2 PD patients with dysphagia. Since then, more reports reinforced the discoveries of pathological α-syn in the ENS (Wakabayashi et al., 1988, 1990; Braak et al., 2006). Braak et al. (2003) postulated that LBs were first localized in the dorsal motor nucleus of the vagus (DMNV) and then spread to the upper brain regions, which inspired more researches on the time course of the presence of LBs in the gut and brain. Stokholm et al. (2016) found that phosphorylated α-syn positive profiles were seen in 22 of 39 (56%) prodromal PD subjects and 30 of 67 (45%) prodromal tissue blocks, which were significantly higher compared to control subjects. Rota et al. (2019) found that the gastrointestinal α-syn pathology precedes in the CNS at least by 6 month in human-A53T α-syn transgenic mice. These evidences consolidate the view that PD patients and animal models of PD are characterized by enteric α-syn pathology at the early stages of the disease. Therefore, it has been proposed that PD may be initiated from the gut (Braak et al., 2004).

Crohn’s disease (CD) and ulcerative colitis (UC) are the two main inflammatory bowel diseases (IBD) with UC mainly affecting the colon and rectum, and CD impacting the small and large intestine (Baumgart and Carding, 2007; Baumgart and Sandborn, 2007). Coincidentally, IBD shares some features with PD, including a negative correlation with smoking (Hernán et al., 2001, 2002; James, 2003; Breckenridge et al., 2016; Kridin et al., 2018; Salih et al., 2018; Wang et al., 2018) and several shared risk genes, such as LRRK2 (Miyake et al., 2010; Hui et al., 2018) and CARD15 (Bialecka et al., 2007). Interestingly, Devos et al. (2013) reported the inflammatory responses in the gut since the early stages of PD, by analyzing the ascending colonic biopsies of PD patients, they found that the pro-inflammatory cytokines (TNF-α, IF-γ, IL-6, and IL-1β) and glia markers GFAP and Sox-10 were significantly elevated, and some of them (IL-6, IL-1β, and Sox-10) were negatively correlated with disease duration. Cote et al. (2011) treated MYD 88 knockout mice with MPTP intraperitoneal administration, they found that MYD 88 knockout mice protected against MPTP induced TH-immunoreactive neuron degeneration in the myenteric plexus of distal ileum, although they detected no macrophage density changes compared with MPTP treated WT mice, the MYD 88 knockout mice exhibited a predominant pro-repair phenotype. Moreover, Cote et al. (2015) found clear presence of M1 monocytes and increased IL-1βand IL-6 in the gut, while in the partial depletion of M1 monocytes the mice protected against MPTP induced TH expression in the gut but not in the striatum, in the meanwhile the microglia activation showed no difference in microglia activation in the brain. In addition, several studies have reported the causal relationship between IBD and PD in recent years (Lin et al., 2016; Fujioka et al., 2017; Wan et al., 2018; Zhu et al., 2019). Here, we analyzed the recent studies on the relationship between PD and intestinal disorders, and highlighted the potential underlying mechanisms of gut inflammation triggering PD.

## Relationship Between Gut Inflammation and PD

In the earlier years, most of the findings were supportive of a relationship between PD and IBD. Lin et al. (2016) demonstrated that IBD was associated with an increased incidence of PD, especially in CD, in a retrospective clinical cohort from 2000 to 2011. In a Danish nationwide cohort study (1977–2014), Villumsen et al. (2019) also found that patients with IBD had a 22% increased risk of developing PD, compared to non-IBD individuals. However, the increased risk of PD was significantly higher in the patients with UC, but not significantly different among patients with CD. These findings were questioned by Weimers et al. (2018), however, after a thorough re-examination, the same conclusions were drawn (Villumsen et al., 2018). In contrast to these favorable reports, some studies have challenged the view on the association between PD and IBD. Fujioka et al. (2017) identified only 2 patients with CD among 876 PD patients, which was comparable to the incidence in the general population. Moreover, PD was even inversely associated with either CD or UC, in some of the newly diagnosed PD cases (Camacho-Soto et al., 2018). Although Weimers et al. (2019) found that IBD was associated with a higher risk of PD, the correlation appeared to be caused by a surveillance bias (Weimers et al., 2018).

In a systematic review and meta-analysis, Wan et al. (2018) found that IBD patients did not show increased risk of PD, however, a subgroup analysis showed a significant difference in the more aged patients (>60 years old). Zhu et al. (2019) suggested that both CD and UC patients have an increased risk of PD compared to the control subjects. To date, although no consensus has been reached, among all of the researches, the most recent data implied that IBD exacerbates PD (Peter et al., 2018). However, the underlying mechanisms on how IBD could trigger PD pathogenesis are still unclear.

## Evidence of Gut Inflammation Spreading to the Brain

As we hypothesized, gut inflammation was able to trigger PD symptoms, the three key factors, (1) the initiator from gut inflammation, (2) the pathways, and (3) the subsequent effects of gut inflammation in the brain must be analyzed step by step. In the gut the inflammatory response induces the disrupted intestinal mucosal barrier, resulting in the exposure to microbiota. Thus, the enteric nervous system, immune system, and microbiota interplayed, which is considered to be a mutually integrated interaction network (Yoo and Mazmanian, 2017). Then, the products or stimulation of these comprehensive interactions can spread to the brain, which may summarize as the microbiota-immune-neuro gut-brain axis. Considering that pathologic α-syn was reported as triggers of PD, we analyzed the inflammatory response and microbiota induced α-syn pathology in the gut. In addition, the inflammatory response itself was also estimated as the pathological process. Therefore, for the pathways, we will mainly introduce (1) the systemic inflammatory routes that spread proinflammatory factors and (2) the pathologic α-syn propagation pathway.

### The Crosstalk Among Microbiota, Inflammatory Response and Pathological α-Syn in the Enteric Microenvironment

Although researches have demonstrated that anti-inflammatory treatment is effective to ease PD symptoms (Gagne and Power, 2010; Thome et al., 2015, 2016), there is a lack of evidence of gut inflammation induced pathologic α-syn directly. However, increased inflammation in the gut, can increase gut permeability, thereby leaking intruders from the gut lumen, such as microbiota and their metabolites, may trigger aggregation of α-syn (Chorell et al., 2015; Bhattacharyya et al., 2019). Interestingly, the dysbiosis in PD is widely reported (Scheperjans et al., 2015; Cassani et al., 2015) and the fecal microbiota transplant is proven to ease symptoms of PD (Ananthaswamy, 2011), suggesting an important role of microbiota in the initiation of PD. A growing body of evidence highlight the potential role of microbiota inducing pathologic α-syn (Fitzgerald et al., 2019). The first proposed mechanism is conceived from the gut lumen harbors *Escherichia coli* which can produce Curli, an extracellular bacterial amyloid protein. In a study conducted by Chen et al. (2016) rats exposed to curli-producing bacteria displayed increased neuronal α-syn deposition in both gut and brain compared to rats exposed to either mutant bacteria unable to synthesize curli, or to vehicle alone. They also found that α-syn-expressing *Caenorhabditis elegans* fed on curli-producing bacteria expressed enhanced α-syn aggregation (Chen et al., 2016). The key element of Curli, CsgA, contains amyloidogenic peptide repeat motifs shared by human and yeast prions (Cherny et al., 2005; Evans et al., 2015), indicating that the α-syn deposition induced by curli may result from the cross-seeding effects between with α-syn and CsgA in the gut (Chen et al., 2016). Besides, the chaperon-like proteins CsgC and CsgE encoded by the Curli operon are also found to modulate α-syn amyloid formation (Chorell et al., 2015). Another mechanism is that α-syn aggregation could be induced by lipid structure in lipopolysaccharide (LPS) (Bussell and Eliezer, 2003), one of the triggers of PD (Qin et al., 2007), which is mainly in the outer membrane of gram-negative bacteria. By semi-quantitative analysis using nuclear magnetic resonance (NMR), Bussell and Eliezer (2003) proved that unbroken helical α-syn structure adopted an unusual, slightly unwound, α11/3 helix conformation to bind the lipid surface, indicating that α-syn can bind to the lipid structure of LPS. More substantial evidence supports this theory. Kim et al. (2016) found that α-syn monomers after being incubated with LPS, showed a strong thioflavin T fluorescence. When these α-syn fibrils were injected into the striatum of mice (C57BL/6J), phosphorylated-α-syn pathology was found throughout the brains, including the striatum, SNpc, amygdala, and auditory cortex (Kim et al., 2016), demonstrating that LPS induced α-syn fibrils are toxic. Besides, in the study of Bhattacharyya et al. (2019) LPS was found to modulate the overall aggregation kinetics of α-syn in a concentration-dependent manner.

The microbiota is also involved in the inflammatory responses (Hooper et al., 2012). It was reported that *Bacteroides fragilis*, can induce colitis through the activation of STAT3 and Th17 response (Wu et al., 2004) via the NF-κB pathway, which leads to an increase of IL-8 production by intestinal epithelial cells (Wu et al., 2009). Prindiville et al. (2000) described that 19.3% of IBD patients with active disease have enterotoxigenic *B. fragilis* in their stool specimen, while control subjects did not show this subset of bacteria. However, the changes of *Bacteroides* in PD is still of controversy: Hasegawa et al. (2015) reported a decrease in PD patients, while Keshavarzian et al. (2015) reported an increase. The inconsistent reports may result from the different stages of the patients. In a 2-year follow-up study, Minato et al. (2017) reported that the deteriorated group (worsening of UPDRS I scores) had lower counts of Bifidobacterium, *B. fragilis*, than the stable group at year 0 but not at year 2. Besides, the intestinal inflammation was reported to promote the overgrowth of Enterobacteriaceae (Lupp et al., 2007; Zeng et al., 2016), of which the *E. coli* strains is able to induce IL-1β through NLR family pyrin domain containing 3 (NLRP3) -dependent mechanism in PD patients (De la Fuente et al., 2014). Moreover, the Prevotellaceae that reported to be decreased in both intestinal inflammation and PD, is also involved in the inflammation. Prevotellaceae is one of main source of short chain fatty acids (SCFAs), which is reported to provide primary energy for intestinal epithelial cells to maintain the stability of the intestinal barrier, contribute to the development of colonic regulatory T (Treg) cells to limit local inflammation and engage the G protein-coupled receptor GPR43 on neutrophils to diminish their infiltration into tissues (Maslowski et al., 2009; Smith et al., 2013; Morrison and Preston, 2016).

### Neural Pathway of Pathologic α-Syn Spreading

After intensive examination of tissues from the peripheral nervous system and the brain, Braak and his colleagues proposed that the α-syn pathologies may be initiated from the olfactory system and low brain stem, which is connected to the peripheral tissues, such as the gut, via the vagal nerve. Pathological studies with postmortem tissues from the brain and the peripheral tissues suggest that pathological α-syn nucleation and aggregation may occur in the enteric neurons of the gastrointestinal tract and can propagate from the gut to the brain (Braak et al., 2003). According to this hypothesis, LBs may first be initiated in the gut (Braak et al., 2006) and then spread via the vagal nerves to the DMNV, locus coeruleus (LC), substantia nigra (SN) and cortex in sequence (Braak et al., 2003). We and others have provided direct evidence of this route (Holmqvist et al., 2014; Uemura et al., 2018). We injected a human PD brain lysate containing different forms of α-syn or different aggregated forms of recombinant α-syn into the intestinal wall of Sprague Dawley rats and found that the exogenously delivered human α-syn could be rapidly transported via the vagal nerve and reach the DMNV in the brainstem in a time-dependent manner (Holmqvist et al., 2014). Uemura et al. (2018) found phosphorylated α-syn-positive LB-like aggregates in the DMNV 45 days after α-syn preformed fibrils (PFFs) were injected into the mouse gastric wall. Although these studies did not examine whether the pathological a-syn in the DMNV were of exogenous or endogenous origin, they could support a prion-like spreading of a-syn whereby α-syn replicates through a mechanism of self-propagating conformation and assembles into filaments, which can act as a seed to recruit the soluble form of the protein and enhance filament load through the seed extension (Goedert et al., 2010; Hansen and Li, 2012). Very recently, Kim et al. (2019) further reinforced the evidence for gut-to-the-brain α-syn pathology spread (Kim et al., 2019). After injecting α-syn PFFs into the muscular layer of the mouse duodenum and pylorus, the authors detected phosphorylated α-syn in DMNV, LC, SNpc and up to upper brain regions (Kim et al., 2019). More importantly they observed dopaminergic degeneration and motor and non-motor behavioral deficits in response to α-syn PFF injections in the gut (Kim et al., 2019). Vagotomy, which interrupts the spreading of α-syn PFFs from the gut the brain, alleviated the severity of morphological and behavioral alterations (Kim et al., 2019). Interestingly, no pathology was observed when the recipient mice are in α-syn knock-out background (Kim et al., 2019). This study provides strong evidence that α-syn pathology can be spread from the gut to the brain via the route of the vagal nerve, can induce neuronal degeneration, and can cause respective neuronal dysfunction and behavioral defects.

## Systemic Inflammatory Routes

Not only can pathological α-syn induced by intestinal inflammation be transmitted to the brain, but also the inflammatory response itself in the gut can influence the brain. The inflammatory response in the gut may affect the brain through two routes, the neuroimmune pathway and the humoral pathway.

### Neuroimmune Pathway

The neuroimmune pathway of gut inflammation transmitting to brain is mainly conducted by the vagal nerve. It was reported that vagotomized mice and rats presented attenuated social exploration and depression in social investigation induced by intraperitoneal injection of recombinant rat IL-1β (Bluthe et al., 1996a, b). Besides, vagal nerve stimulation could decrease the inflammatory response and improve survival in experimental sepsis, hemorrhagic shock, ischemia reperfusion injury, and other conditions of cytokine excess (Borovikova et al., 2000; Johnston and Webster, 2009; Huffman et al., 2019).

Up to now, the specific mechanisms of the neuroimmune pathway are not clear, but some studies have suggested that the vagal nerve plays a dual role in inflammatory regulation both through its afferent and the efferent fibers (Bonaz et al., 2017). The vagal afferents target the hypothalamic-pituitary-adrenal (HPA) axis. Vagal afferents activate neurons from the A2 noradrenergic group in the nucleus tractus solitarius (NTS). These neurons project to the parvo-cellular paraventricular nucleus of the hypothalamus (PVH), where the corticotrophin-releasing factor (CRF) neurons are stimulated to release CRF, which in turn induces the pituitary to release adrenocorticotropic hormone (Bonaz et al., 2016). Adrenocorticotropic hormone then stimulates the adrenal glands to release glucocorticoid, which plays a role in the inhibition of peripheral inflammation (Bonaz et al., 2017). In addition, Lubbers et al. (2010) showed that activating cholecystokinin-1 receptors on vagal afferents can also regulate inflammation. The vagal efferents are involved in the cholinergic anti-inflammatory pathway. This pathway regulates systemic inflammation through the release of acetylcholine (Ach) by the vagal nerve (Zhai et al., 2017). The details of this pathway remain obscure, but studies have showed that a subunit of the α7 nicotinic Ach receptor (α7nAchR), one of the Ach receptors, is expressed on macrophages, and the α7nAchR-agonist, GTS, is able to restrain systemic inflammation (Wang et al., 2003; Cai et al., 2009).

### Humoral Pathway

Humoral pathways of gut inflammation spreading to brain are mainly involved in the leakage of the Blood–Brain Barrier (BBB), which can be divided into disruptive and non-disruptive approaches, respectively, reflecting the physical conditions of the BBB (Varatharaj and Galea, 2017).

Disruptive BBB change is usually evident in the structural alterations, and can be detected using inserted tracers (Varatharaj and Galea, 2017). A considerable number of reports have shown that the BBB is damaged in PD patients (Pisani et al., 2012; Gray and Woulfe, 2015). In addition, 60% of LPS induced PD models exhibited disrupted BBB (Varatharaj and Galea, 2017). In the event that the blood-brain barrier is damaged, proinflammatory cytokines and immune cells such as T cells (Engelhardt and Ransohoff, 2012) and mast cells (Jones et al., 2019) from peripheral inflammation are able to enter the brain.

Non-disruptive BBB changes usually occur at a molecular level, and are not visible in histological architecture (Varatharaj and Galea, 2017). The changes can be mediated by special transporters (Xaio et al., 2001; Osburg et al., 2002; Hartz et al., 2006; Pan et al., 2008; Jaeger et al., 2009; Wittmann et al., 2015), cytokines (Herkenham et al., 1998; Skelly et al., 2013), prostaglandins (PGs) (Vasilache et al., 2015) and cellular transmigration (Bohatschek et al., 2001; Wang et al., 2008; Banks et al., 2012). In addition, substances can also enter the brain through the areas that lack the BBB, such as the circumventricular organs (Ferrari and Tarelli, 2011). Therefore, proinflammatory cytokines such as IL-1α, IL-1β, IL-6, TNF-α, etc., can enter the brain and activate microglia or astrocytes to induce inflammatory response (Vallieres and Rivest, 1997; Osburg et al., 2002; Sato et al., 2012; Neniskyte et al., 2014).

## Subsequent Effects of Gut Inflammation in the Brain

Pathological α-syn, which could be triggered by intestinal bacterial components, is the main component of LB, whose neurotoxicity is related to its structure and post-translational modification, such as phosphorylation at Ser129 (Arawaka et al., 2017) and nitration (He et al., 2019). These pathological proteins, on the one hand, recruit normal α-syn and disrupt its physiological functions; on the other hand, their toxicity can act as environmental stress to increase inflammation, oxidative stress, and interfere with other physiological effects (Xu et al., 2013; Zhang et al., 2017). Another subsequent effect is that the immune cells and proinflammatory cytokines in the brain can cause additional release of inflammatory and neurotoxic molecules, contributing to chronic neuroinflammation and neuronal death (Ferrari and Tarelli, 2011; Tufekci et al., 2012). The underlying mechanisms of these subsequent effects inducing neurodegeneration are unclear, however, they may be involved in a series of molecular mechanisms.

It should be emphasized that the pro-inflammation and pathological α-syn propagation may not work alone, they are interrelated both in the brain and the gut. For example, marmosets with colitis show significantly increased phosphorylated α-syn in the colonic myenteric ganglia (Resnikoff et al., 2019). In the brain, fibrillar α-syn is found to produce pro-inflammatory mediators, such as IL-1β. IL-1β via the activation of the microglial NLRP3 inflammasome (Codolo et al., 2013; Gordon et al., 2018). α-Syn is also found to activate pro-inflammatory TLR4 pathways in astrocytes (Rannikko et al., 2015) and disrupt the anti-inflammation of Dopamine D2 receptor (Du et al., 2018). In contrast, in a study conducted by Horvath et al. (2018) pro-inflammatory factor S100A9 is also reported to induce α-syn aggregation.

### Oxidative Stress

Oxidative stress occurs when excessive oxygen free radicals are produced within cells. When the concentration of these active substances is not controlled by internal defense mechanisms, such as antioxidants or oxygen free radical removal enzymes, protein lipids, and DNA are oxidized causing damage (Gagné, 2014). Studies have shown that the production of high levels of reactive oxygen species (ROS) and reactive nitrogen species (RNS) and the reduction of antioxidant substances lead to neuronal cell death in neurodegenerative diseases (Farooqui and Farooqui, 2009; Melo et al., 2011).

Oxidative stress in the brain is usually found along with inflammatory responses such as activated immune cells, cytokines and other inflammatory mediators (Leszek et al., 2016). These inflammatory responses activate microglia, then microglial activation with gliosis results in an oxidative burst, which releases ROS, including superoxide anion (O_2_^–^), hydrogen peroxide (H_2_O_2_), the highly reactive hydroxyl radical (HO^⋅^) and RNS such as nitric oxide (NO) into the environment (Tufekci et al., 2012). In addition, NO can react with O_2_^–^, producing peroxynitrite (ONOO^–^), which is a powerful oxidant and may decompose to form HO^⋅^ (Melo et al., 2011).

Pathological α-syn is also reported to be involved in causing oxidative stress in a large number of reports (Esteves et al., 2009; Renella et al., 2014; Deas et al., 2016; Perfeito et al., 2017; Russo et al., 2019). Oxidative stress-induced toxicity depends on the structures of α-syn. Deas et al. (2016) found that although both oligomeric and fibrillar α-syn can induce free radicals, only oligomeric forms of a-syn cause neurotoxicity and endogenous glutathione reduction. They also found that oligomer-induced ROS depends on the presence of metal ions, because the addition of metal chelators can block oligomer-induced ROS and reduce neuronal death (Deas et al., 2016). Interestingly, α-syn also mediates oxidative stress caused by metal ions: down-regulation of the α-syn protein significantly increases cell viability and reduced oxidative stress in maltose-aluminated cells (Saberzadeh et al., 2016). Therefore, the oxidative stress induced by metal ions or α-syn, is probably the result of the two co-factors.

### Glutamate Excitotoxicity

Glutamate is the main cognitive neurotransmitter in the brain, inducing an excitatory response when binding to its receptors (Dong et al., 2009). In order to maintain a high signal-to-noise ratio outside the synapse, the extracellular concentration of glutamate is required to be very low. The overspill of glutamate and excessive activation of glutamate receptors can lead to neuronal dysfunction and cell death, known as excitatory toxicity (Dong et al., 2009).

Pathological α-syn has been shown to affect all of the glutamate receptors: N-methyl-D-aspartic acid (NMDA), α-amino-3-hydroxy-5-methylisoxazole-4-propionate (AMPA) and kainic acid (KA) receptors (Dong et al., 2009). Firstly, prolonged exposure to α-syn oligomers are reported to increase basal synaptic transmission through NMDA receptor activation, triggering enhanced contribution of calcium-permeable AMPA receptors (Diogenes et al., 2012). Secondly, nanomolar concentrations of large α-syn oligomers, formed by incubating α-syn with organic solvent and Fe (3+) ions, altered both pre- and post-synaptic mechanisms of AMPA-receptor-mediated synaptic transmission (Huls et al., 2011). Furthermore, Chang et al. (2012) found that melatonin attenuates KA-induced neurotoxicity through the reduction of KA-increased α-syn aggregation, which indicates that KA-induced neurotoxicity may be mediated by α-syn aggregation. Thus, pathological α-syn may lead to the activation of ionotropic receptors, contributing to glutamate excitotoxicity.

It has also been widely demonstrated that inflammation can induce glutamate excitatory toxicity. Firstly, monocyte-derived macrophages and activated microglia are able to induce glutamate excitotoxicity, which may result from their function of extruding glutamate into the extra synaptic space in exchange for cystine via the cystine/glutamate exchanger (Xc) – transporter (Kigerl et al., 2012). Secondly, the astrocyte function of clearing, buffering and containing glutamate abilities can be decreased by inflammatory factors, such as TNF-α, IL-1β, and IF-γ (Haroon and Miller, 2017; Haroon et al., 2018). In addition, immune activation can increase glutamate-like molecular, quinolinic acid, which (1) over excites the NMDA receptor, (2) inhibits glutamine synthetase, a critical enzyme in the glutamate-glutamine cycle in astrocytes, keeping the stable glutamate level and (3) promotes glutamate release (Guillemin, 2012).

### T-Cell Driven Inflammation

The central nervous system was thought to be isolated from the adaptive immune system for a long period of time (Carson et al., 2006). However, Louveau et al. (2015) discovered the lymphatic system in the brain of mice, which were verified in monkeys and humans thereafter (Absinta et al., 2017). In addition, inflammatory factors from peripheral inflammation, such as IL-1β, TNF-α can also act on the blood–brain barrier to allow peripheral lymphocytes to enter the brain.

It is well established that, T cells, especially the CD4+ T-cells, are involved in PD. Brochard et al. (2009) found a significant increase of T-cells but not B-cells in post-mortem brains of PD patients. Later, they identified that lacking CD4+ cells, but not CD8+ cells, led to an attenuated response to MPTP-induced dopaminergic cell death in mice (Brochard et al., 2009). Additionally, Reynolds et al. (2010) identified that the subsets of CD4+ cells, T-helper-1 (Th1) and T-helper-17 (Th17), are the main causers of MPTP-induced cell death. The mechanism by which gut inflammation causes PD through CD4+ T-cells can be seen. First of all, peptides derived from two regions of α-syn (the Tyr 39 and phosphorylated Ser129 region) can act as antigenic epitopes (Sulzer et al., 2017). Therefore, pathological α-syn can be captured at the lymph node, and be presented to CD4+ T-cells by antigen presenting cells (APCs) (Campos-Acuña et al., 2019). If naïve CD4+ cells are activated, they transform into their subtypes: Th1 and Th17. When the BBB is damaged, Th1 and Th17 infiltrate the brain, where the microglia act as local APCs, presenting α-syn antigen via MHC II, polarizing CD4+ T-cells to the Th1 and Th17 subtypes (Campos-Acuña et al., 2019). The Th1 and Th17 cells produce a large number of inflammatory factors, such as, TNF, IFN-γ, IL-1, IL-2, and IL-21 (Kaiko et al., 2008), which in turn re-stimulate glial cells (M1 microglia) to produce large amounts of glutamate, inflammatory factors, ROS and RNS (Gonzalez et al., 2015), thereafter recruiting leukocytes from the blood and exaggerating more inflammatory reactions (Mosley et al., 2012).

### COX-2

Cyclooxygenase-2 (COX-2), also known as prostaglandin-endoperoxide synthase 2, is one of the three cyclooxygenases (COX-1, COX-2, and COX-3) (Teismann, 2012) that primarily induces the synthesis of prostaglandins from arachidonic acid (Kirkby et al., 2016). COX-2 is mainly detected in distal dendrites and dendritic spines, especially in excitatory neurons (Kaufmann et al., 1997). In general, COX-2 is not detected in dopaminergic structures such as the SN and striatum (Teismann et al., 2003). However, in PD patients and MPTP-induced mouse models, the immunopositive reaction of COX-2 in dopaminergic neurons is intense (Teismann et al., 2003). Therefore, there exists a high possibility that COX-2 may be related with dopaminergic neuron death.

The mechanisms through which COX-2 damages neurons may be through two mechanisms. (1) The first one is via oxidative stress. Arachidonic acid can be converted to PGH_2_ in two steps. Firstly, arachidonic acid reacts with 2O_2_ to form the prostaglandin G_2_ (PGG_2_). Secondly, PGG_2_ is converted by the cyclooxygenases to form prostaglandin H_2_ (PGH_2_) (Smith et al., 2000). The second step of COX-2 induction reacts far more rapidly than COX-1, however, the COX-1 reaction involves the reduction of two electrons of superoxide substrate, while about 40% of the COX-2 conversion just reduces one electron of superoxide substrate (Teismann, 2012). In this situation, the leaked electrons can react with oxygen to produce reactive oxygen species (Teismann, 2012). (2) Another possible cause of COX-2 induced neuron death is the crosstalk between cytokines and PGs, one of COX-2’s metabolized products (Yao and Narumiya, 2019). Traditionally, PGs are mostly reported to have an inhibitory effect on acute inflammation (Narumiya and Furuyashiki, 2011). However, the expression of COX-2 are widely found in chronic inflammation, such as IBD (Wallace, 2001), rheumatoid arthritis (Mikos, 1976; Jasani and Bach, 1979; Svendsen et al., 1985; Fattahi and Mirshafiey, 2012; Kirkby et al., 2016), and multiple sclerosis (Mirshafiey and Jadidi-Niaragh, 2010). Based on this, studies found that PGs can crosstalk with cytokines and amplify the cytokines’ effects. On the one hand, PGs induce expression of relevant cytokine receptors, which is typically observed in Th1 cell differentiation and Th17 cell expansion (Yao et al., 2009); on the other hand, PGs and cytokines synergistically activate NF-κB to induce expression of inflammation-related genes, including chemokines and COX-2 itself (Yao and Narumiya, 2019). These signals amplify chronic immune inflammation and exacerbate

neuronal death. In addition, the overexpression of COX-2 in dopaminergic neurons also plays a role in α-syn accumulation (Bartels and Leenders, 2010).

## The Reaction of Central Neuroinflammation in Gut

Among the various pre-clinic PD animal models, the intranigral injections of 6-OHDA or LPS directly act on the nigra-striatal system, thus, they could be used to elucidate the effect of nigral-striatal degeneration on the gut, comparing to the peripheral administration which may affect the gut first or at the same time. In the 6-OHDA induced PD model, gastrointestinal dysfunctions have widely been reported (see Table 1), such as delayed gastric empty (Zheng et al., 2011, 2014; Toti and Travagli, 2014; Vegezzi et al., 2014; Feng et al., 2019), impaired gastric motility (Zheng et al., 2011, 2014; Vegezzi et al., 2014; Feng et al., 2019), impaired colonic transit (Fornai et al., 2016; Pellegrini et al., 2017), decreased weight and water content of the feces (Zhu et al., 2012), colonic relaxation defect (Colucci et al., 2012), decreased intraluminal pressure (Colucci et al., 2012), decreased frequency of peristalsis (Colucci et al., 2012). In addition, in the studies of Pellegrini et al., inflammatory evidence is also presented in the model of intranigral injection of 6-OHDA, such as the increased GFAP, TNFα, IL-1β, eosinophils and mast cell in colon. In addition, in the study of intranigral injection of LPS, the impaired gastric motility is also observed. Further, in a study conducted by Ulusoy et al. (2017) the pathological α-syn was transported from the brain to the stomach. Therefore, the central neuroinflammation and nigrostriatal degeneration could, in turn, spread to the ENS and contribute to exacerbated intestinal inflammatory responses and gastrointestinal dysfunction via brain-to-the-gut descending pathways, thus generating a positive loop that could drive the chronicization of the ongoing central and peripheral neuroinflammatory and neurodegenerative processes and contribute to gut motor dysfunctions.

**TABLE 1:** Summary of intestinal function and inflammatory alterations in Parkinson’s disease (PD) animal models of intranigral injection of 6-OHDA or LPS.

**Model (species)**	**Functional evidence**	**Inflammatory evidence**	**References**
Bilateral intranigral injection of 6-OHDA (rat)	Delayed gastric empty; Impaired gastric motility;		Zheng et al., 2011
	Delayed gastric empty; Impaired gastric motility;		Zheng et al., 2014
	Delayed gastric empty and intestinal transport; decreased fecal pellets and content		Feng et al., 2019
Unilateral intranigral injection of 6-ohda (rat)	Delayed gastric empty;		Toti and Travagli, 2014
	Delayed gastric empty; Constipation		Vegezzi et al., 2014
	Decreased weigh and water Content of fecal matter;		Zhu et al., 2012
	Impaired colonic transit;		Fornai et al., 2016
	Impaired colonic transit;	GFAP↑(colon); TNF-α↑(colon); IL-1β↑(colon); Eosinophils↑ (colon); Mast cells↑ (colon)	Pellegrini et al., 2017
	Colonic relaxation defect; Decreased intraluminal pressure; Decreased frequency of peristalsis		Colucci et al., 2012
		TNF↑ (colon); IL-1β↑ (colon); Eosinophils↑ (colon); Mast cells↑ (colon)	Pellegrini et al., 2016
Bilateral intranigral injection of LPS (rat)	Impaired gastric motility		Zheng et al., 2013

## Conclusion

**FIGURE 1:**
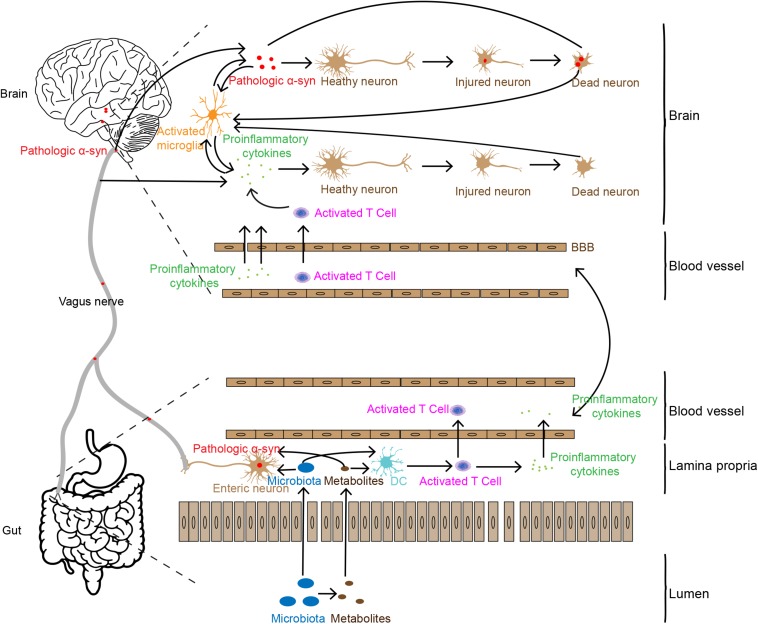
Possible pathways involved in gut inflammation induced neuron death in the brain. Gut inflammation may increase intestinal permeability, allowing the leakage of bacteria and their metabolites which may trigger pathologic α-syn aggregation and pro-inflammatory cytokine production and release in the ENS. Pathologic α-syn is propagated to the brain via the vagal nerve, and inflammatory cytokines are transported to the brain through the humoral pathway or stimulate the vagal nerve to produce pro-inflammatory factors in the brain. Pro-inflammatory cytokines and synucleinopathies in the brain may induce neuronal injury and death, which in turn enhance more severe inflammatory responses.

Based on the analyses above, we summarize the potential association of intestinal inflammation in PD pathogenesis, as shown in Figure 1. Increased intestinal permeability caused by gut inflammation induces the leakage of flora and its metabolites into the body (Berg, 1995; Camilleri et al., 2012; Mu et al., 2017), stimulating the production of pathologic α-syn or pro-inflammatory cytokines (Chen et al., 2016; Kim et al., 2016; Fukui, 2017). Pathologic α-syn can be spread to the brain via the vagal nerve and pro-inflammatory cytokines and immune cells transmit to the brain through the humoral system. In addition, the leakage of flora and its metabolites from gut lumen can also activate immune cells, such as T cells. These immune cells can infiltrate into the brain via the disrupted BBB caused by the pro-inflammatory cytokines. In the brain, the two factors, pathologic α-syn and pro-inflammatory cytokines and immune cells enhance the dysfunction and degeneration of dopaminergic neurons. They, together with tissue debris or diseased proteins released from lysed cells, trigger the cascade and feedback loop of inflammation, including microglial activation, and neuronal dysfunction and cell death.

Of note, although a large body of evidence has implied the relationship between PD and gut inflammation, details on how the process takes place are still largely unknown. Further investigations are required to clarify the mechanisms of the mutual transformation between intestinal inflammation, microbiota and pathological α-syn or other PD-related pathogens.

Overall, we have briefly addressed the mechanisms on how gut inflammation is transmitted to the brain and how it causes damage in the brain. We hope that this will provide some clues for further studies in this interesting field of research.

## Author’s Note

This material has not been published in whole or in part elsewhere. The manuscript is not currently being considered for publication in another journal. All authors have been personally and actively involved in substantive work leading to the manuscript, and will hold themselves jointly and individually responsible for its content.

## Author Contributions

Q-QC and J-YL conceived of the review and drafted the manuscript. CH and WL edited the manuscript. All authors approved the manuscript.

## Conflict of Interest Statement

The authors declare that the research was conducted in the absence of any commercial or financial relationships that could be construed as a potential conflict of interest.
